# Tarlov Cysts Misdiagnosed as Adnexal Masses in Pelvic Sonography: A Literature Review

**DOI:** 10.3389/fmed.2020.577301

**Published:** 2020-11-30

**Authors:** Shengshu Kim, Ho jun Lee, Joong Hyun Park, Taeyeon Kim, Kiyeun Nam

**Affiliations:** ^1^Department of Physical Medicine and Rehabilitation, Dongguk University Ilsan Hospital, Dongguk University College of Medicine, Goyang-si, South Korea; ^2^Department of Neurology, Inje University Sanggye Paik Hospital, Inje University College of Medicine, Seoul-si, South Korea

**Keywords:** perineural cyst, Tarlov cyst, sclerotherapy, management, prognosis

## Abstract

**Introduction:** A Tarlov cyst (TC) is a perineural cyst filled with cerebrospinal fluid that originates from the dorsal ganglion or the spinal posterior nerve root. TCs are usually asymptomatic and incidentally found in the sacral region. Endopelvic extension of TCs is uncommon and can be misdiagnosed as an adnexal mass on gynecological ultrasound imaging.

**Methods:** We performed a search for all clinical studies of TCs that mimicked adnexal masses that had been published through October 12, 2020. We placed no restrictions on language or year of publication in our search, and we performed searches with the following keywords: perineural cyst, Tarlov cyst, sclerotherapy, management, and prognosis. We included all misdiagnosed cases or cases considered as adnexal masses on pelvic sonography.

**Results:** We identified 21 cases of TCs mimicking adnexal masses and conducted a comprehensive analysis of these 21 cases to assess the epidemiology, symptoms, initial diagnoses, provisional ultrasound diagnoses, confirmative modalities, sizes, locations, treatments, and outcomes. The 21 cases included 16 symptomatic cases (76%) and 5 cases with incidental findings (24%), and the average patient age was 41.3 years. The initial diagnosis was performed with ultrasonography in all cases. The most frequent misdiagnosis was unspecified adnexal mass. Confirmative diagnostic modalities were MRI only (67%), CT only (5%), and both MRI and CT (28%). Treatments were surgery (33%), conservative treatment (19%), percutaneous intervention (5%), and alcohol sclerotherapy (5%). In two symptomatic cases misdiagnosed as pelvic masses, cystectomy was performed and leakage of cerebrospinal fluid occurred, necessitating repair of the leak. In one of the asymptomatic patients, cauda equina syndrome occurred after alcohol sclerotherapy for misdiagnosed TC. However, the patient improved with no neurologic deficit after 18 months of conservative treatment.

**Conclusion:** The possibility of large TCs should be considered when assessing adnexal masses in sonography. Since TCs can masquerade as pelvic masses, they should be considered if the mass appears tubular/cystic or multilocular/multiseptate, does not move with respiration, and originates from the sacrum in sonography with or without neurologic symptoms. Accurate diagnosis can prevent medical mismanagement and reduce patient discomfort.

## Introduction

Tarlov cysts (TCs) were first described by Dr. Tarlov in 1938 (1) as perineural cysts filled with cerebrospinal fluid (CSF) that originate from the dorsal ganglion or the spinal posterior nerve root (2). TCs are usually found in the sacral region, are usually asymptomatic, and usually present as incidental finding (3). According to the literature, about 1% of TCs are symptomatic (4). The symptoms of TCs include low back pain, radiculopathy, leg weakness, and paresthesia in the lower limb (5). Magnetic resonance imaging (MRI), computed tomography (CT), or myelography can be used to confirm TC, with MRI being the gold standard modality (6). Electromyography (EMG) can be useful for assessing patients who show symptoms of neurologic changes (4). There is no consensus on the appropriate treatment of TCs; known treatments for symptomatic TCs include conservative management, percutaneous interventions, or surgical procedures such as laminectomy and fenestration (7). Although endopelvic extension of TCs is uncommon, these cysts may present as an incidental finding on routine gynecological ultrasound imaging (8). Gynecologists and obstetricians who lack experience with large TCs may often misdiagnose these cysts as adnexal masses.

**Figure 1:**
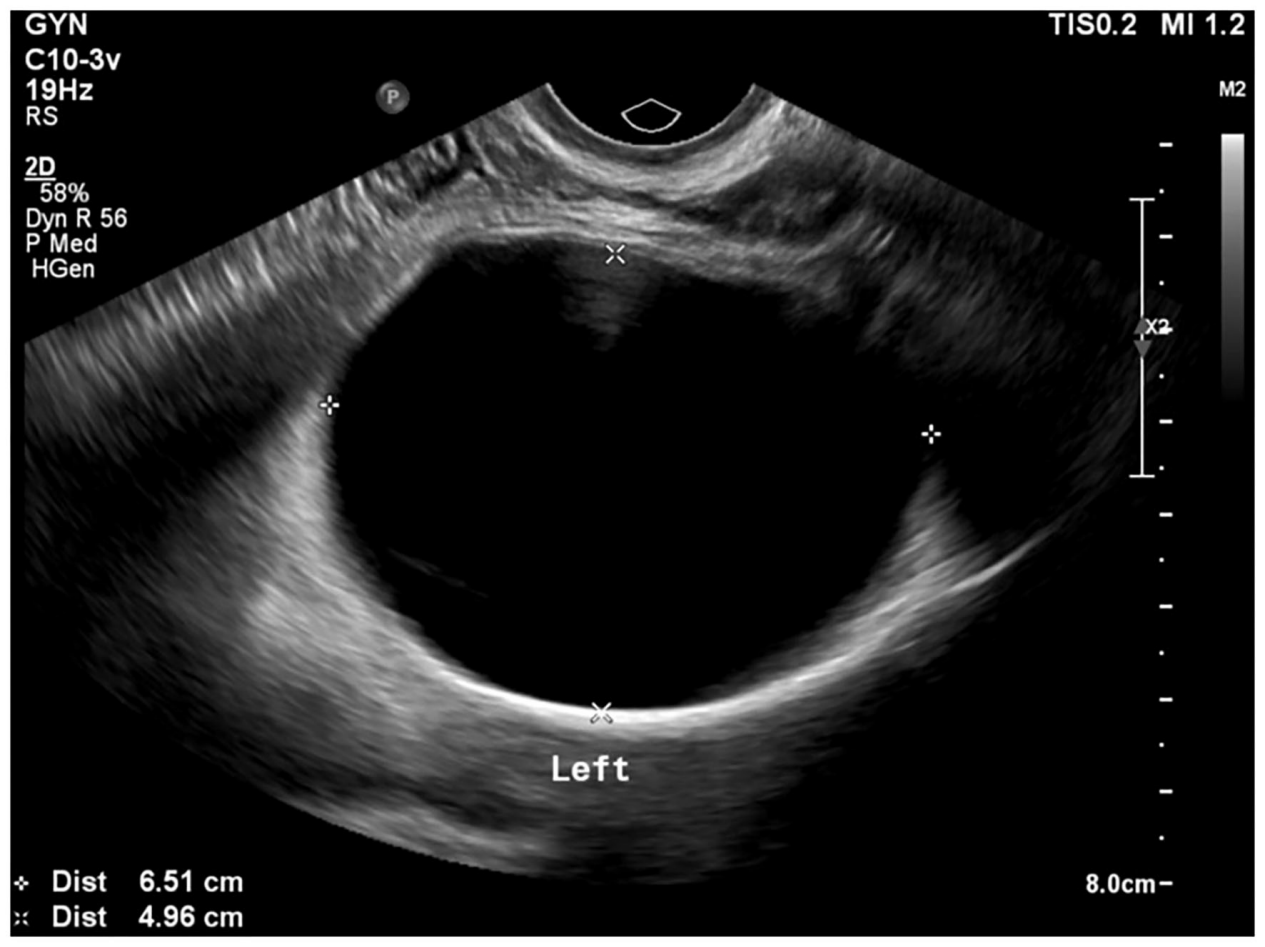
Pelvic ultrasonography shows a left 6.51 × 4.96 cm ovarian cyst.

**Figure 2:**
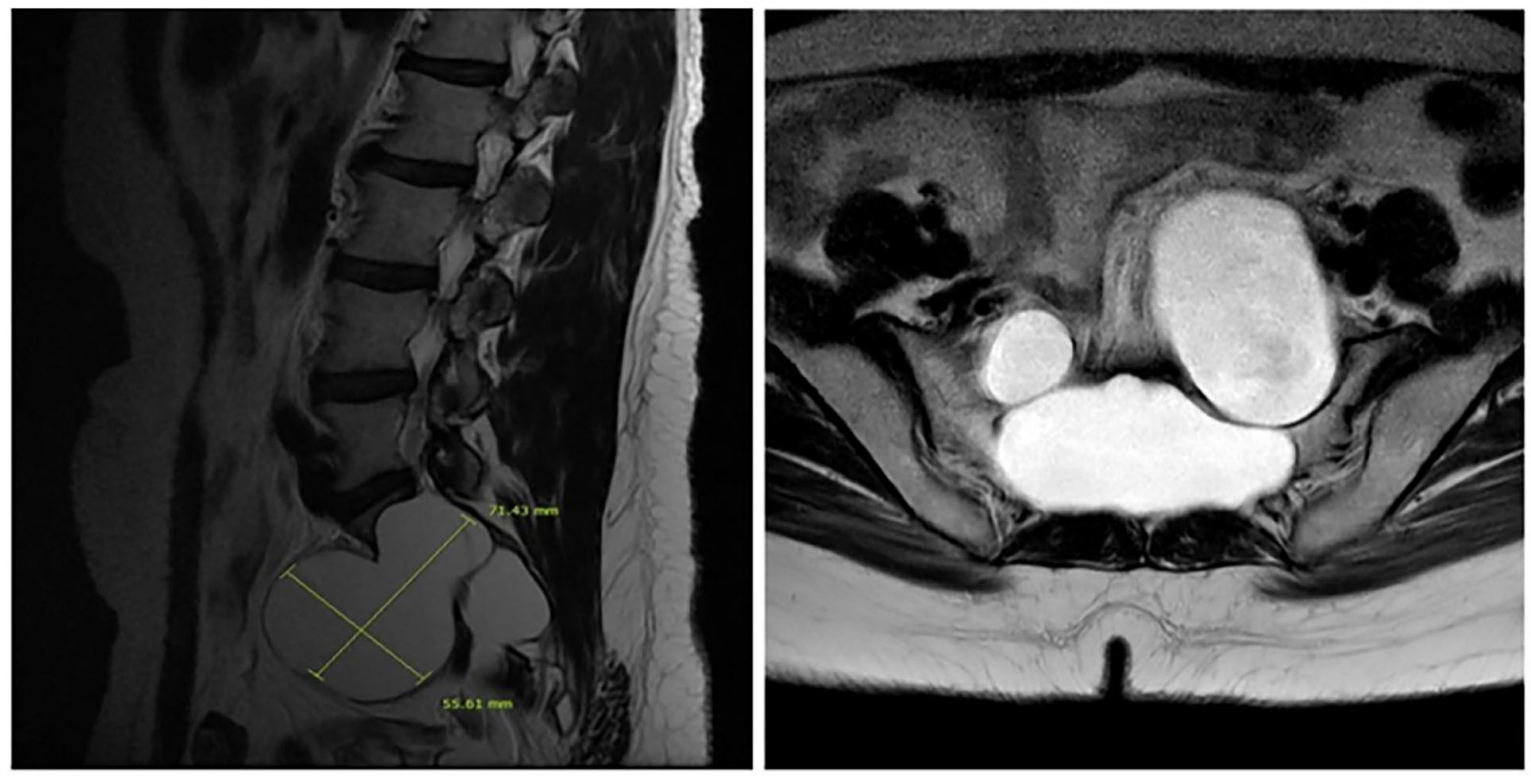
A sagittal T2-weighted image shows a 7.1 × 5.6 cm cyst that originated from the perineurium in the sacrum with extension to the pelvic cavity with bony erosion (left) and the sacrum on the transverse T2-weighted image (right).

**Figure 3:**
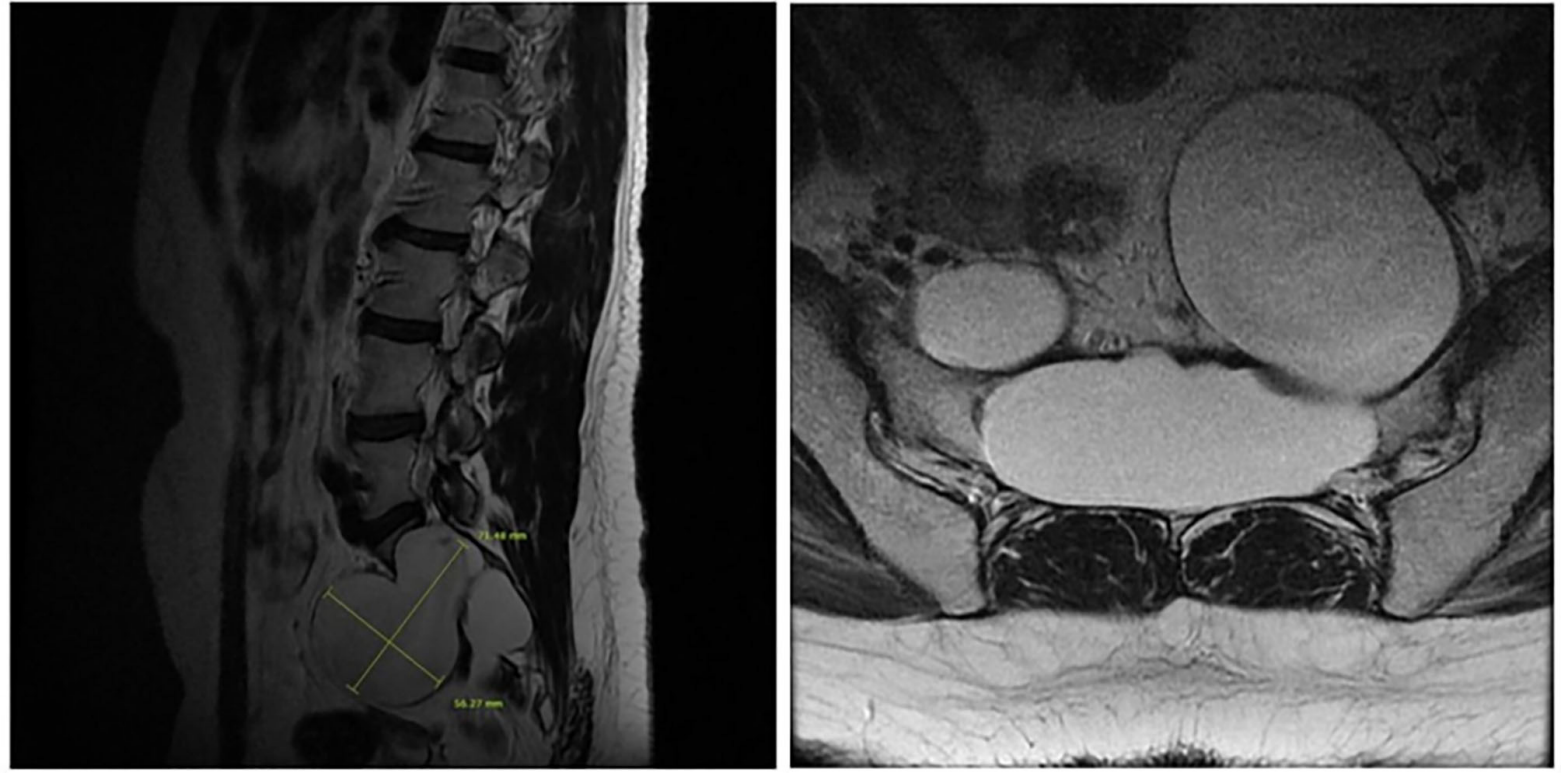
A sagittal T2-weighted image shows a 7.1 × 5.6 cm cyst that originated from the perineurium in the sacrum with extension to the pelvic cavity with bony erosion (left) and the sacrum on a transverse T2-weighted image (right) obtained 10 months after sclerotherapy.

We report a case in which a TC was misdiagnosed as an adnexal cyst in a 38-year-old woman (gravida 1, para 1). She had visited a local gynecology hospital for a routine health checkup without any symptoms, and pelvic ultrasonography showed a left 6.51 × 4.96 cm cystic mass (Figure 1). The lesion was an anechoic unilocular cyst with a smooth thin wall (no internal septations or solid component). The patient was premenopausal and her pregnancy plan was not clear. The cyst was relatively large and had grown in size by ~1 cm over a year. The clinician diagnosed the cyst as a simple ovarian cyst and planned to remove it. Instead of surgical treatment, alcohol sclerotherapy was selected to preserve ovarian function and ensure patient convenience. Sclerotherapy was performed with a fine needle under transvaginal sonographic guidance. Under direct US guidance, the needle was inserted through the vaginal fornix into the center of the cyst, and the fluid was aspirated. Subsequently, 100% ethanol injection and irrigation was conducted using 20 mL of ethanol twice. After sclerotherapy, the patient experienced severe headache when upright, which was accompanied by nausea and vomiting as well as bilateral lower leg weakness and radiating pain in the left leg. Because her symptoms persisted after 1 week, she visited a local medical spinal center. Spine MRI showed a 7.1 × 5.6 cm cystic mass originating the sacral area (Figure 2). Brain MRI was also performed and it showed no abnormal lesions. The patient was treated with medication for pain control and physical therapy. After conservative treatment, the headache, nausea, and vomiting improved, but the weakness in both legs and radiating pain in the left leg persisted. Two months after sclerotherapy, the patient first visited the department of physical medicine and rehabilitation at our hospital by walking independently. On the Medical Research Council manual muscle test, muscular weakness was observed in both lower limbs (grade 3+ on the right side and 3 on the left side; Table 1). She complained of bilateral sensory change below the L4 dermatome, manifesting as pain, temperature, vibration, and proprioception. Ankle clonus and Babinski sign were negative. Deep tendon reflex was slightly decreased at the knee and ankle jerk, and the anal tone was decreased. The patient also complained of anal sphincter weakness of 40% and reported symptoms of a neurogenic bladder, namely, incontinence, nocturia, and high urinary frequency; however, self-voiding was possible. To determine the cause of the weakness, pain, and sensory change, and ascertain the severity and localization, we performed electrophysiological examinations. It revealed lower lumbosacral polyradiculopathy and sacral arc dysfunction, clinically cauda equina syndrome (CES) (Table 2). We applied therapeutic modalities to the left leg to relieve the radiating pain, and the patient received therapeutic exercises which include lower leg muscle stretching, strengthening, and balancing training. After 6 months, the lower leg weakness, radiating pain in the left leg, urinary incontinence, anal sphincter weakness, and sensory change showed some improvement. Follow-up EMG revealed that the CES was in an incomplete recovery state (Table 2), while follow-up spine MRI showed no change in the size 10 months after sclerotherapy (Figure 3). The radiating pain in the left leg disappeared and muscle strength normalized after 18 months (Table 1). Moreover, the symptoms of neurogenic bladder disappeared and anal sphincter function normalized. On follow-up EMG, CES was in nearly full recovery state (Table 2).

**Table 1:** MRC grades 2, 6, and 18 months after alcohol sclerotherapy.

**MRC grade (Rt/Lt)**	**2 months**	**6 months**	**18 months**
Hip flexor	3+/3	5/4	5/5
Knee extensor	3+/3	5/4	5/5
Ankle dorsi flexor	3+/3	4/3+	5/5
Hallucis extensor	3+/3	4/3+	5/5
Ankle plantar flexor	3+/3	4/3+	5/5

*MRC, Medical Research Council; Rt, right; Lt, left*.

**Table 2:** Needle EMG findings 2, 6, and 18 months after alcohol sclerotherapy.

	**Spontaneous activity**					**MUAP**			**Recruit pattern**
**Muscle**	**IA**	**Fib**	**PSW**	**Fasc**	**CRD**	**Amp**	**Dur**	**PPP**	
**2 MONTHS**									
Lt. TA	N	None	None	None	None	N	N	N	C
Lt. GCM	N	2+	2+	None	None	N	N	N	R/S
Lt. PL	N	2+	2+	None	None	N	N	+	R/S
Lt. VM	N	None	None	None	None	N	N	N	R/C
Lt. TFL	N	None	None	None	None	N	N	N	R/C
Lt. S1	N	None	None	None	None	–	–	–	–
Lt. G-max	N	None	None	None	None	N	N	N	R/C
Lt. sphincter	N	3+	3+	None	None	N	N	+	R
**6 MONTHS**									
Rt. TA	N	None	None	None	None	N	N	N	R/C
Rt. GCM	N	None	None	None	None	N	N	N	R/C
Lt. GCM	N	None	None	None	None	N	N	+	R
Lt. PL	N	None	None	None	None	N	N	+	R
Lt. sphincter	N	2+	2+	None	None	N	N	+	R/C
Rt. sphincter	N	2+	2+	None	None	N	N	N	C
**18 MONTHS**									
Lt. GCM	N	None	None	None	None	N	N	N	R/C
Lt. sphincter	N	None	None	None	None	N	N	+	R/C
Rt. sphincter	N	None	None	None	None	N	N	+	R/C

*Rt., right; Lt., Left; TA, tibialis anterior; GCM, gastrocnemius; PL, peroneus longus; VM, vastus medialis; TFL, tensor fascia lata; S1, S1 paraspinal muscle; G-max, gluteus maximus; IA, insertion activity; N, normal; Fib, fibrillation potential; MUAP, motor unit action potential; PSW, positive sharp wave; Fasc, fasciculation; CRD, complex repetitive discharge; Amp, amplitude; Dur, duration; PPP, polyphasic potential; C, complete; R/S, reduced to single; R/C, reduced to complete; R, reduced*.

## Materials and Methods

Written informed consent was obtained from the patient for the publication of any potentially identifiable images or data included in this article.

### Literature Search Strategy

We reviewed all papers that reported TCs mimicking adnexal masses, with the papers identified by searching MEDLINE, EMBASE, SCOPUS, Web of Science, the Cochrane Central Register of Controlled Trials, the World Health Organization International Clinical Trials Registry Platform, and the clinical trials registry and database of the U.S. National Institutes of Health (ClinicalTrials.gov) through October 12, 2020. We placed no restrictions on language or year of publication in our search, and we performed searches using the following keywords: Tarlov cyst, perineural cyst, adnexal mass, sclerotherapy, management, and prognosis.

We identified 21 patients with TCs mimicking adnexal masses in 12 studies (Table 3). We included all cases in which the TC was misdiagnosed or considered as an adnexal mass in pelvic sonography, and we excluded cases in which the TC was not misdiagnosed or considered as an adnexal mass.

**Table 3:** Clinical data of patients with TCs mimicking adnexal masses.

**Case**	**References**	**Age/** **Sex**	**Main symptoms**	**Duration of** ** symptom (years)**	**Modality of** ** provisional diagnosis**	**Initial diagnosis**	**Modality used** ** for confirmation**	**Location**	**Cyst size** ** (cm)**	**Follow up** ** (months)**	**Interve-ntion** ** or surgery**	**Outcome**
1	Raza et al. (9)	54/F	Asymptomatic	(-)	US	Pelvic mass	CT, MR	S (L, R)	5	7	N	(-)
2	Maleci et al. (8)	29/F	Asymptomatic	(-)	US	Presacral mass	MR	S2 (L)	NA	NA	Y	Improved
3	Mark et al. (3)	42/F	Perineal pain and bowel disturbance	NA	US	Adnexal mass	MR	NA	NA	NA	NA	NA
4		43/F	Left lower quadrant pain and dysmenorrhea	NA	US	Adnexal mass	MR	NA	NA	NA	NA	NA
5		28/F	Pelvic pain, dysmenorrhea, and dyspareunia	NA	US	Adnexal mass	MR	NA	NA	NA	NA	NA
6		41/F	Pelvic pain	0.2	US	Adnexal mass	CT, MR	NA	NA	NA	NA	NA
7		44/F	Lower abdominal pain	NA	US	Adnexal mass	CT, MR	NA	NA	NA	NA	NA
8	Ishii et al. (5)	29/F	Constipation and low back pain	NA	US	Presacral mass	MR	S3 (L)	11	NA	Y	Improved
9	H'ng et al. (2)	29/F	Pelvic pain and difficulty ambulating	1	US	Hydrosalpinges	MR	S1 (R, L)	6.4; 6.2	3	N	Improved
10		29/F	Lower abdominal pain	NA	US	Ovarian cyst	CT	S1 (L)	7.1	NA	N	Improved
11		26/F	Asymptomatic	(-)	US	Hydrosalpinx	MR	S2 (R, L)	7.1; 4.1	NA	N	(-)
12	Hirst et al. (10)	76/F	Poor urinary flow	NA	US	Ovarian cyst	MR	NA	5	NA	Y	Unchanged
13	Saboo and Di Salvo (6)	54/F	Asymptomatic	(-)	US	Adnexal mass	CT, MR	S1, S2, and S3 (L, R)	7.5; 6.1	NA	N	(-)
14	Wang et al. (11)	67/F	Pelvic pain, explosive diarrhea, and dysuria	8	US	Presacral mass	CT, MR	S2 (R)	6.7	3	Y	Improved
15	Firoozeh et al. (12)	45/F	Pelvic pain	NA	US	Hydrosalpinges	MR	S2 and S3 (L, R)	NA	3	N	Unchanged
16	Wang et al. (13)	32/F	Sacrococcygeal pain and left leg pain	0.5	US	Presacral mass	CT, MR	S1 and S2 (L, R)	NA	96	Y	Improved
17		30/F	Right lower quadrant pain	2.5	US	Pelvic mass	MR	S2 (R)	NA	18	Y	Improved
18	Monique et al. (14)	49/F	Lower abdominal discomfort	1	US	Adnexal mass	MR	S1, S2, S3, and S4	6.3	12	N	NA
19	Present case	38/F	Asymptomatic	(-)	US	Ovarian cyst	MR	S1 (L)	7.1	18	N	Improved
20	Hanshuo et al. (15)	47/F	Low back pain, right leg pain, and conspitation	10	US	Presacral mass	MR	S1-5 (R, L)	7.1	6	Y	Improved
21		36/F	Low back pain and right groin pain	0.5	US	Pelvic mass	MR	S2-5 (L, R)	6.3	5	Y	Improved

*NA, non available information; MR, magnetic resonance image; CT, computed tomography; S, sacral level; R, right; L, left; N, no; Y, yes*.

## Results

We identified 21 cases of TCs mimicking adnexal masses and analyzed the epidemiology, symptoms, initial diagnoses, provisional ultrasound diagnoses, confirmative modalities, sizes, locations, treatments and outcomes in the selected cases.

### Epidemiology and Clinical Manifestations

The median patient age was 41 years (range: 26–76 years, mean: 41.3 years, standard deviation: 13.04 years). All patients were female, and 16 were symptomatic (76%). Pelvic pain (5 cases, 24%) (2, 3, 11, 12) and low abdominal pain (5 cases, 24%) (2, 3, 13, 14) were the most common symptoms, followed by low back pain (15) and perineal pain (3, 13) (2 cases each, 10%) and constipation (5) and low urinary flow (10) (1 case each, 5%). Five patients were asymptomatic (24%) (2, 6, 8, 9). The duration of symptoms was approximately 1 year.

### Imaging Study

The initial diagnostic modality was ultrasonography in all cases. The most common incorrect diagnosis or initial diagnosis was an unspecified adnexal mass (3, 6, 14). Other diagnoses included presacral mass (5, 8, 11, 13, 15), hydrosalpinges (2, 12), ovarian cyst (2, 10), and pelvic mass (9, 13, 15). Confirmative diagnostic modalities were MRI only (14 cases, 66.7%) (2, 5, 8, 12–15), both MRI and CT (6 cases, 28.6%) (3, 6, 9, 11, 13), or CT only (1 case, 4.8%) (2). All cysts were sacral lesions that showed pelvic extension. The average cyst size was 6.9 cm [range: 5 cm (9, 10) to 11 cm (5), median: 6.9 cm, standard deviation: 1.46 cm].

### Management

The patients were treatments with surgery (7 cases, 33%) (5, 8, 10, 11, 13, 15), conservative management (4 cases, 19%) (2, 12, 14), percutaneous intervention (1 case, 5%) (13), alcohol sclerotherapy (1 case, 5%), and observation (3 cases, 14%) (2, 6, 9) (Table 4). Limited treatment-related information was available for five cases (24%) (3).

**Table 4:** Type of management depending on the presence of symptoms in TCs misdiagnosed as adnexal masses and the clinical course.

	**Management**		**Course**
Symptomatic (*n* = 16)^†^	Conservative treatment (*n* = 4)		Improved in 2 cases (2) Unchanged in 1 case (10) Non-available information in 1 case (12)
	Intervention (*n* = 1, Percutaneous drainage and fibrin injection)		Improved (11)
	Surgery	Laminectomy (*n* = 3)	Improved in 2 cases (5,9) Unchanged in 1 case (14)
		Cystectomy (*n* = 2)	Aggravated (CSF leak, improved after repair surgery) (11,13)
		Fistula blocking and cyst filling (*n* = 1)	Improved (13)
Asymptomatic (*n* = 5)^††^	Intervention (*n* = 1, Alcohol sclerotherapy)		Aggravated (Cauda equina syndrome, improved after physical therapy)
	Surgery (*n* = 1, Cystectomy)		Aggravated (CSF leak, improved after repair surgery) (8)

† *Of 16 cases, The number of non-available information cases was five (3)*.

†† *Of 5 cases, The number of observation cases was three (2, 6, 9)*.

*n, the number of cases*.

Five patients were asymptomatic. Among them, one underwent unnecessary surgery (8), one (the case described above) received alcohol sclerotherapy, and the other three (2, 6, 9) did not receive any treatment (Table 4).

The types of surgery were laminectomy in three cases (5, 10, 11), repair of leakage after cystectomy in three cases (8, 13, 15), and fistula-blocking surgery in one case (15). Conservative treatment consisted of administration of a nonsteroidal anti-inflammatory drug, physical therapy, and antibiotics. In the one case that involved a percutaneous intervention (13), the patient underwent percutaneous drainage and fibrin glue injection. Unusually, one non-symptomatic case was treated with alcohol sclerotherapy (100% alcohol irrigation, 20 mL, immediately repeated twice). The time to intervention or surgery was within 1 month. In the cases in which symptoms improved after conservative treatment, the duration of treatment was several months.

### Prognosis

Among the three symptomatic patients who underwent laminectomy performed on symptomatic patients, two showed pain relief (5, 11) and one showed no improvement (10). In the patient who underwent fistula-blocking and cyst-filling surgery, the TC shrank and the patient reported relief from symptoms (15). Two (2) of the four patients who received conservative treatment showed symptom improvement, one (12) showed no change in symptoms, and no information was available for the fourth patients (14). The patient who received percutaneous drainage and fibrin glue injection (13) showed symptom improvement within 3 months and cyst shrinkage over 96 months of follow-up (Table 4).

In one case (8), an asymptomatic large TC was misdiagnosed as a presacral mass, and marsupialization was performed with exploratory laparotomy, resulting in leakage of CSF; the patient experienced headache, vomiting, and sixth cranial nerve palsy. A reoperation was performed to ligate the CSF fistula, and the patient's symptoms fortunately improved. In the other two symptomatic cases misdiagnosed as pelvic masses (13, 15), cystectomy was performed and a CSF leak occurred, requiring repair of the leak; the symptoms improved after repair surgery. In our case, incorrect treatment with alcohol sclerotherapy of an asymptomatic large TC mistaken for an adnexal mass caused chemical CES. With conservative treatment, symptoms improved without significant complications after 18 months. Thus, these three previous cases (8, 13, 15) and our case presented unexpected complications as a result of mistaking the TC as a pelvic mass or adnexal mass.

## Discussion

In the present case, the clinician performed alcohol sclerotherapy after a misdiagnosing a TC as a simple ovarian cyst. Alcohol sclerotherapy is a transvaginal ultrasound-guided aspiration and ethanol injection technique that is presently used as an alternative therapeutic modality for simple ovarian cysts or endometriomas (16, 17). The clinician chose alcohol sclerotherapy to preserve ovarian function and to ensure for patient convenience. However, since the patients actually had a TC, not a simple ovarian cyst, the alcohol sclerotherapy caused chemical CES. The anechoic unilocular cyst should have been diagnosed more carefully. In pelvic sonography, the clinician could have checked the separation of the cyst from the ovary, its immobility with respiration, and its connection to the sacrum to identify the TC. For more appropriate diagnosis and treatment, MRI would have been helpful. According to the consensus statement of the Society of Radiologists in Ultrasound, MRI should have been performed, not an immediate treatment (18). However, unfortunately, immediate treatment was implemented in this case. Nevertheless, the patient showed nearly full recovery without significant complications after 18 months. This clinical course also provides important information regarding the regeneration after alcohol-induced denervation in TC.

Our literature review outlines the importance of familiarizing gynecologists and obstetricians to TCs, which are cystic masses distinct from functional cysts, endometriomas, teratoma, hydrosalpinges, and peritoneal inclusion cysts with similar characteristics (19). TCs show a tubular/cystic or multilocular/multiseptate appearance in pelvic sonography (2). However, these features may not be apparent in all cases, and TCs can show various manifestations ranging from a simple rounded cyst to a complex loculated cystic mass. Because TCs can show various sonographic features, the following aspects should be considered together.

- The size of the TCs that were mistaken for adnexal masses in the literature review was 5 cm or more (mean size: 6.9 cm), while the median age of the population was 41 years. Accordingly, clinicians should consider the possibility of TCs when treating middle-aged women with adnexal cysts that are 5 cm or greater in size.
- Since TCs are extraperitoneal cysts, they do not show mobility during respiration.
- Since TCs are perineural cysts originating from the lumbosacral nerve root, identification of a connection to the posterior pelvic wall is important, i.e., TCs are located posteriorly on the sacrum.
- In contrast to other adnexal cysts, TCs are separated from reproductive organs such as the ovary and salpinx.
- Patients presenting with a symptomatic endopelvic TC may show low back pain, sciatica, leg weakness, and other neurologic deficits, so these findings may also serve as a point of differentiation.

These differentiating points are summarized in Table 5. TCs can also have internal echoes and appear as slightly elongated, multilocular, or beaded cystic masses posteriorly (12). If an observed cyst is posteriorly located and does not move with respiration, TCs should be considered along with abscesses, hematomas, endometriomas, and lymph nodes (2). Although an endometriotic cyst with adhesion could show low mobility with respiration, extraovarian endometriosis is rarely cystic and, in general, does not reach a large size (3). TCs appear less elongated and tubular than hydrosalpinges, and the incomplete septation or “waist sign” observed in hydrosalpinges may not be present in a TC (20). The presence of ovarian tissue with follicles around the mass will help confirm an ovarian origin (12).

**Table 5:** Differential diagnosis of benign cystic adnexal masses.

	**Sonographic features**	**Mobility with** **respiration**	**Connection to** **sacrum**	**Connection to** **reproductive organ**	**Symptoms**
Functional (physiologic) cyst	-Simple cyst -Thin walled and unilocular cystic mass	High	No	Yes	Asymptomatic
Endometrioma	Low-level internal echoes, mural echogenic foci, or nonvascular solid attenuating components	High (unless adhesion)	No	Yes	Pelvic pain Dysmenorrhea Dyspareunia Infertility
Teratoma	Echogenic attenuating component or small horizontal interfaces	High	No	Yes	Asymptomatic
Hydrosalpinx	Tubular cystic mass with or without folds	High	No	Yes	Asymptomatic Pelvic pain Infertility
Peritoneal inclusion cyst	Cystic masses taking the shape of the underlying space located adjacent to or surrounding a functioning ovary	High	No	Yes	Pelvic pain Asymptomatic
Tarlov cyst	-Deep cystic masses communicating with sacral foramina -Vary from a simple rounded cyst to a complex loculated cystic mass with septations	Low	Yes	No	Pelvic pain Low back pain Perineal pain Sciatica Leg weakness

A comprehensive understanding of TCs, including the points of differentiation, begins with a thorough understanding of anatomy. TCs are perineural cysts of the sacral area that arise between the perineurium and endoneurium. In 1938, Dr. Tarlov first reported a TC of the filum terminale as an incidental autopsy finding, and the lesion was classified as a type II meningeal cyst by Nabors et al. (21). TCs are cysts filled with CSF and are usually located near the dorsal root ganglion and can contain nerve fibers (4). Since their original description, TCs have been found all along the spinal nerve roots, not only in the lumbar region (10). One study revealed the following incidence rates for perineural cysts at the different spinal levels: cervical level, 1.18%; thoracic, 5.53%; lumbar, 1.05%; and sacral, 15.17% (22). Among the cases showing TCs, single anomalies were found in 29% and multiple unilateral or less frequently bilateral changes were noted in the remaining 71% (22). The prevalence of TCs is estimated to be between 1 and 5% of the population, and 20–26% of TCs are thought to be symptomatic, accounting for about 1% of the population (4). The prevalence of TCs increases with age (23). They are significantly more common in women, and women are also more likely to be symptomatic (4). MRI studies of patients with back pain have revealed that 70% of patients with TCs are women, and sex-related differences in the composition of the dura mater or spinal nerve roots have been postulated to be the underlying cause of this female predominance (7).

The pathogenesis of these cysts is unclear, although various hypotheses have been proposed to explain the formation of the slit valve mechanism that allows CSF to pass into the cysts (10). There is however causal evidence supporting traumatic hemorrhage, pseudomeningoceles, hydrostatic CSF pressures, congenital diverticula from persistent embryonic fissures, inflammation in the subarachnoid space, inflammation within the nerve root cysts leading to inoculation of fluid, arachnoidal proliferation along and around the exiting sacral nerve root, and hemosiderin deposits breaking down venous drainage in the epineurium and perineurium after trauma (4). Consequently, the temporarily increased pressure within the cyst may stretch any overlying nerve fibers within the cyst wall or may compress the ventrally displaced main portion of the nerve root, which, in turn, may lead to exacerbated radiculopathy or sensory loss, compression of the adjacent sacral thecal sac, and associated urinary and bowel incontinence (24). Symptomatic cysts do not resolve (25). In a retrospective cohort study of 28 subjects, TCs showed relative growth rates of 2.9 ± 2.6%, 4.3 ± 3.8%, and 1.4 ± 1.4% in the anteroposterior, craniocaudal, and transverse dimensions per year, and none of the cysts decreased in size between successive MRI examinations (26). Bone erosion is quite common and is a characteristic feature of large TCs that have grown quickly (5). Although it is unclear whether bone erosion causes bony pain, rapid growth of the TC may result in bony pain. In the slit-valve mechanism, TCs can grow gradually, and TCs up to 11.3 × 10.3 × 9 cm in size have been reported (5).

Common clinical presentations include low back pain, sacrococcygeal pain, leg weakness or pain, sciatica, perianal pain, neurogenic claudication, bowel and bladder dysfunction, and sexual disturbances. TCs can also cause unusual clinical symptoms (abdominal or pelvic pain) if the cysts are in the presacral region (13). MRI, CT, or myelography can be used to confirm the findings of TCs, with MRI being the gold standard modality (6). TCs are isodense with CSF on noncontrast CT scans and can often be seen to cause various osseous abnormalities and erosions (27). In MRI, TCs showed high signal intensity on T2-weighted sequences and low signal intensity on T1-weighted sequences (12). MRI can also be used to delineate the exact relationship of the cyst to the thecal sac, as well as the total volume of fluid within the cyst (27).

Asymptomatic TCs generally do not require treatment (27). Conservative treatment, including medical therapy and physical therapy, is suggested as the first-line option for symptomatic TCs (21). Nonsteroidal anti-inflammatory drugs and neuropathic pain medications have been shown to yield mild improvement in pain symptoms (4), and oral steroids have been reported to be helpful in the treatment of TCs (21). Pelvic physical therapy may help alleviate any associated pelvic floor myofascial pain or dysfunction (4). Epidural steroid injections have also been shown to be helpful in treating the radiculopathy associated with TCs, and they may be especially helpful in treating the pelvic pain caused by TCs (28). Other intervention options include external CSF drainage, percutaneous cyst drainage, and percutaneous fibrin glue injection (5). Fibrin deposition on cyst walls impedes CSF ingress, triggers fibrosis, and, ideally, promotes cyst contracture (29). In the gynecological background, simple ovarian cysts can be treated using ethanol (sclerotherapy) to destroy the epithelial lining of the fluid-secreting walls, thereby obliterating the cyst cavity and preventing the re-accumulation of fluid (16). The guidelines for sclerotherapy for adnexal cysts have not been established. Alcohol is neurotoxic and has neurolytic effects that can lead to nerve damage, and therefore should not be injected into TCs. Nevertheless, alcohol sclerotherapy does not cause permanent irreversible or complete nerve damage. In such cases, a serial nerve conduction study and EMG can provide much information about neural regeneration. Patients with cysts >1.5 cm in size and radicular pain or bowel/bladder dysfunction have been reported to benefit from surgery (5). Surgical treatment of symptomatic perineural cysts, which involves complete cyst removal and excision of the affected posterior root and ganglion, was advocated by Tarlov and has since been used by others (30). Surgical options include insertion of cyst–subarachnoid, cyst–peritoneal, or lumboperitoneal shunts; simple decompressive laminectomy; resection of the cyst neck; cyst wall resection; cyst imbrication; or bipolar cauterization to shrink the size of the cyst (5). One study suggested a simple and effective procedure with the key step of blocking the inlet of the fistula from inside the dural sac, which is more applicable and minimizes the probability of cyst recurrence (15). The complications of interventional or surgical treatment for TCs can be quite significant, including cerebral fat embolisms, positional headaches, CSF leaks, aseptic meningitis, postoperative pseudomeningoceles, and damage to the sacral nerve roots, with resultant lower motor neuron bladder or bowel dysfunction (4). As such, interventional or surgical treatment should be carefully considered.

Our study had a few limitations. The primary limitation was the lack of cases, with only 21 relevant cases identified in the literature search. Although misdiagnosed TCs for adnexal masses are not common, we performed a search for all clinical studies of TCs that mimicked adnexal masses. Secondly, the data were obtained retrospectively. Thus, additional research is needed to prevent misdiagnosis and enable more accurate diagnosis and treatment in the future.

## Conclusions

Our data indicates the importance of considering the possibility of a large TC when assessing adnexal masses on sonography. Since TCs can masquerade as pelvic masses, if the masses appear tubular/cystic or multilocular/multiseptate, do not move with respiration, and originate from the sacrum in sonography with or without neurologic symptoms, TC should be considered. Our case is the first to report chemical CES caused by alcohol sclerotherapy for a TC that was incorrectly diagnosed as an adnexal mass. However, the patient recovered almost completely without significant complications after 18 months. Accurate diagnosis can prevent incorrect medical management and reduce patient discomfort.

## Data Availability Statement

The original contributions presented in the study are included in the article/supplementary materials, further inquiries can be directed to the corresponding author/s.

## Ethics Statement

Written informed consent was obtained from the individual(s) for the publication of any potentially identifiable images or data included in this article.

## Author Contributions

SK and TK: data analysis, data interpretation, literature search, and writing of the manuscript. HL and JP: conceptualization, methodology, and writing of the manuscript. KN: supervision, project administration, and review of the manuscript. All authors contributed to the article and approved the submitted version.

## Conflict of Interest

The authors declare that the research was conducted in the absence of any commercial or financial relationships that could be construed as a potential conflict of interest.
